# VHL-Associated Optic Nerve Hemangioblastoma Treated with Stereotactic Radiosurgery

**DOI:** 10.15586/jkcvhl.2018.104

**Published:** 2018-06-06

**Authors:** Hiroshi Kanno, Seiki Osano, Masamichi Shinonaga

**Affiliations:** 1 Department of Neurosurgery, International University of Health and Welfare School of Medicine, Narita, Japan; 2 Department of Neurosurgery, International University of Health and Welfare Atami Hospital, Atami, Japan; 3 Department of Neurosurgery, Shonan Fujisawa Tokushukai Hospital, Fujisawa, Japan

**Keywords:** optic nerve hemangioblastoma, stereotactic radiosurgery, VHL, von Hippel-Lindau disease

## Abstract

Central nervous system hemangioblastomas are generally restricted to the cerebellum, spinal cord, and brainstem. Supratentorial hemangioblastomas are uncommon, and optic nerve hemangioblastomas are extremely rare, with fewer than 25 reports including this case. In this report, we present the case of a 36-year-old woman with von Hippel–Lindau (VHL) disease who presented with progressive diminution of vision in the left eye due to a retrobulbar optic nerve hemangioblastoma. The patient had a history of cerebellar/spinal hemangioblastomas and pancreatic cysts, and her father and brother were patients with VHL disease. Gadolinium-enhanced magnetic resonance imaging showed intraorbital retrobulbar–enhanced mass on the left optic nerve. The optic nerve hemangioblastoma was treated with fractionated stereotactic radiosurgery using Novalis. Eighteen months after the stereotactic radiosurgery, the tumor volume decreased although the patient lost vision. This report presents an extremely rare case of optic nerve hemangioblastoma, which is the first case treated with stereotactic radiosurgery.

## Introduction

Von Hippel–Lindau (VHL) disease is an autosomal dominant disorder originating from aberrations of the *VHL* tumor suppressor gene that was identified on chromosome 3p25 (1). The incidence of VHL is estimated to be 1 in 36,000 to 1 in 43,000 live births (2, 3, 4). VHL disease is classically associated with renal cell carcinomas, renal cysts, pancreatic islet cell tumors, pheochromocytomas, and endolymphatic sac tumors (5). Central nervous system (CNS) hemangioblastoma is the most common VHL-associated lesion, and it is found in 70–80% of VHL patients. Hemangioblastoma is a WHO grade I benign tumor that does not metastasize to remote organs but is associated with significant neurologic morbidity and mortality based on its location and multiplicity (6). In VHL patients, 50–60% of the hemangioblastomas are located in the cerebellum, 40–50% in the spinal cord, 10–20% in the brain stem, and 2–4% in the pituitary stalk. In the spinal cord, 30–50% are located in the thoracic segments, 40–50% in the cervical segments, and 10–20% in the lumbar segments (7, 8). CNS hemangioblastoma is the earliest or the second earliest manifestation of VHL patients, and the onset age of CNS hemangioblastoma ranges from 7 to 73 years, with the mean being 29 years (7, 9). Signs and symptoms vary based on the anatomical tumor location, associated edema and cyst, and tumor size. Usually, tumors that become symptomatic and require resection grow faster than the asymptomatic ones (10). Most symptoms caused by hemangioblastomas do not arise from the solid tumor itself but from the associated rapidly growing cyst or syrinx (11). Therefore, symptoms can occasionally develop rapidly, though usually they develop slowly.

Previous large-scale studies on VHL patients have shown that hemangioblastomas have a sporadic growth pattern with periods of growth followed by growth arrest, that is, a saltatory growth pattern (12). Patterns of growth vary and are categorized as saltatory (70–75% of growing tumors), linear (5–7%), or exponential (20–25%). Many tumors will remain the same size for several years (12). In a recent study, VHL patients were found to have a mean of 8.5 tumors/patient (range: 1–33 tumors/patient) at initial evaluation. Mean tumor development was 0.4 new tumors/year and was correlated with age, with more frequent development in younger patients (13).

Regarding neuroradiological findings, hemangioblastomas are most often visualized by contrast-enhanced T1-weighted MRI or contrast-enhanced CT. In post-contrast images, the tumor tissue appears as a homogenous bright contrast-enhanced mass that clearly stands out from the surrounding tissue. T2-weighted or flair MRI allows excellent quantification of edema and peritumoral cysts, which appear as high-signal areas (14).

Hemangioblastomas make up nearly 2% of all intracranial tumors and 10% of posterior fossa tumors in adults (15, 16). Most hemangioblastomas present as posterior fossa masses. Less than 5% of hemangioblastomas present in the supratentorial compartment. Hemangioblastomas have been rarely seen in the pituitary stalk, optic nerves, and the ventricles (17–19). Herein we report a case of an intraorbital optic nerve hemangioblastoma in a patient with VHL, which was treated by stereotactic radiosurgery.

### Case Report

**Figure 1.**
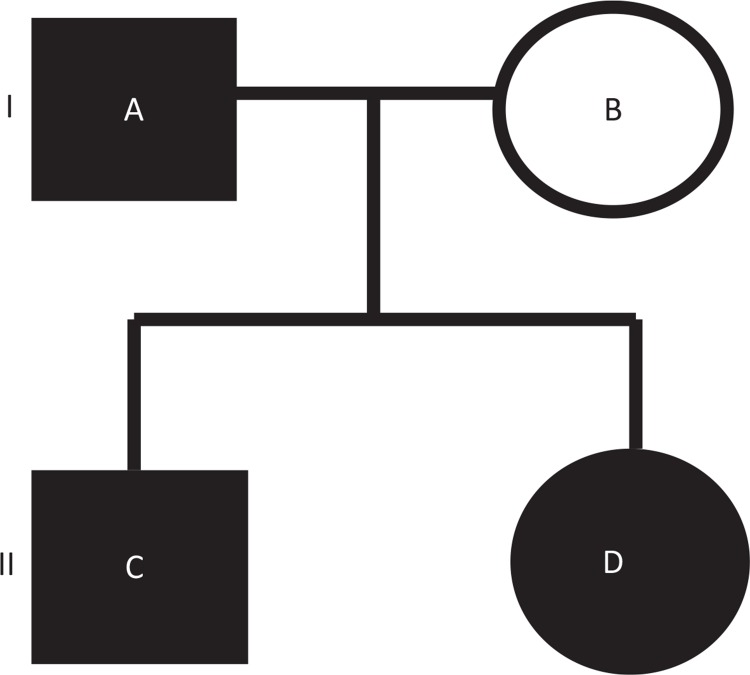
The family pedigree of the patient. (A) The patient’s father is a 65-year-old VHL patient. (B) The patient’s mother is a 64-year-old non-VHL woman with malignant lymphoma and colon cancer. (C) The patient’s brother is a 39-year-old VHL patient. (D) The current VHL patient is a 36-year-old woman with an optic nerve hemangioblastoma.

The patient is a 36-year-old right-handed female with a known history of VHL disease. Her 65-year-old father had been diagnosed with VHL disease and multiple cerebellar hemangioblastomas; her 39-year-old elder brother had also been diagnosed with type 1 VHL disease without pheochromocytoma because of a complete deletion of the *VHL* gene. This family pedigree is shown in Figure 1.

**Figure 2.**
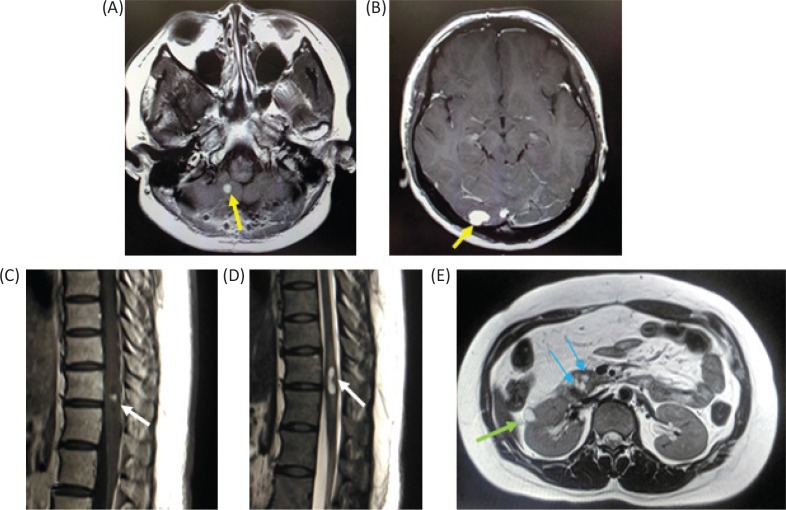
Magnetic resonance imaging of the patient. (A and B) Gadolinium (Gd)-enhanced T1-weighted image of cerebellum when the patient was 26 years old. The patient had multiple cerebellar hemangioblastomas. (C) Gd-enhanced T1-weighted image of the lower thoracic cord. A small hemangioblatoma with a syrinx is shown. (D) T2-weighted image of the lower thoracic cord. (E) T1-weighted image of the abdomen. A renal cyst and multiple pancreatic cysts are shown.

At the age of 26, the patient underwent surgical treatment for cerebellar hemangioblastoma. She also had a spinal cord hemangioblastoma at 11th thoracic vertebra level, a renal cyst and multiple pancreatic cysts (Figure 2).

**Figure 3.**
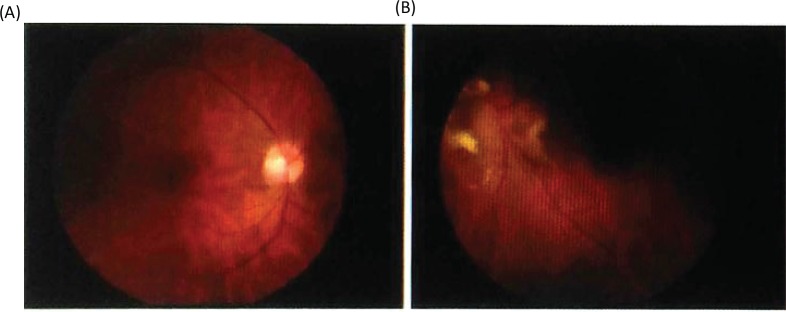
Optic fundi before radiosurgery. (A) Left healthy side. (B) Right lesion side.

When her father underwent surgical treatment for a cystic cerebellar hemangioblastoma at our hospital (International University of Health and Welfare Atami Hospital), she visited our hospital, at the age of 36, because of recently developed visual disturbance. Her visual acuity decreased gradually and her visual filed became narrow. The visual acuities at the first visit to our hospital were 0.1 on the right side and sensus luminis on the left side. The examination of her left ocular fundus showed a pale papilla (Figure 3).

**Figure 4.**
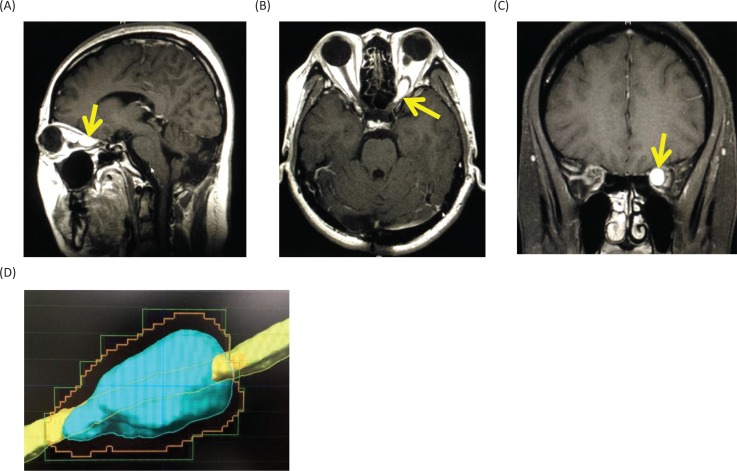
Magnetic resonance images before radiosurgery. (A) Gadolinium (Gd)-enhanced T1-weighted image, sagittal. (B) Gd-enhanced T1-weighted image, axial. (C) Gd-enhanced T1-weighted image, coronal. Yellow arrows show the tumor. (D) Three-dimensional planning MRI for radiosurgery. The light blue color indicates the tumor enveloping the optic nerve.

Magnetic resonance imaging (MRI) of the head showed a Gd-enhanced well-circumscribed mass on the left retrobulbar optic nerve in the orbital, and three-dimensional MRI showed the tumor enveloped the left optic nerve (Figure 4).

**Figure 5.**
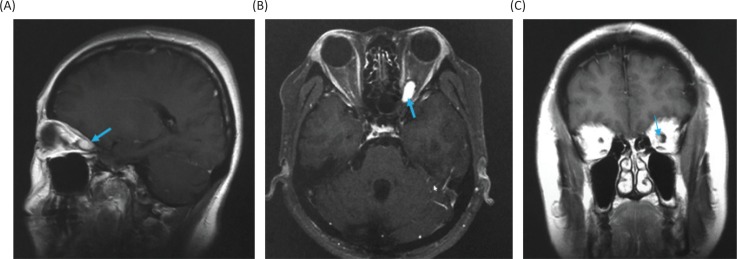
Gadolinium-enhanced magnetic resonance images at 18 months after the stereotactic radiosurgery. (A) Sagittal image. (B) Axial image. (C) Coronal image.

The differential diagnoses for the tumor include optic glioma, optic sheath meningioma, optic nerve schwannoma and optic nerve hemangioblastoma. Among them, since the patient is a VHL patient, the most probable diagnosis was thought to be an optic nerve hemangioblastoma. Therefore, we made a decision to treat the tumor with stereotactic radiosurgery. At Shonan Fujisawa Tokushukai Hospital, the stereotactic radiosurgery was prescribed with the dose being 39 Gy/13 fractions using Novalis. The planning target volume was set at 0.7 mL, and D95 (95% of standard irradiation volume) was set at 35 Gy/13 fractions. The patient completely lost her vision after the radiosurgery. Eighteen months after the radiosurgery, the tumor volume slightly decreased (Figure 5), but her vision has been still lost. The consent to publish the case has been given by the patient.

## Discussion

VHL disease is an autosomal dominant disorder that frequently develops CNS hemangioblastomas. Patients with a complete deletion of the VHL gene have been found to have a decreased incidence of retinal hemangioblastoma when compared with patients with a missense mutation (20). This patient did not have a retinal hemangioblastoma but had an optic nerve hemangioblastoma.

The origin of stromal cell, that is, neoplastic cell of hemangioblastoma, has been suggested to be an embryonic hemangioblast cell which is a common precursor for hematopoietic and endothelial cells (21–23). During normal embryonic development, Scl is only transiently expressed at the embryonic hemangioblast cells in the diencephalon containing optic nerve, metencephalon, and spinal cord. In addition, Scl protein expression is shown in CNS hemangioblastoma in retina, spinal cord, brainstem, and cerebellum. Diencephalon contains optic nerve, and therefore, hemangioblastoma possibly originates from optic nerve (24–26). The striking topographical analogy between embryonic Scl expression and the sites of tumor development may, therefore, help explain the selective topography of CNS hemangioblastoma.

The differential diagnosis for a solitary optic nerve tumor includes an optic nerve sheath meningioma and an optic glioma. Meningioma is commonly associated with neurofibromatosis type 2 (27) and optic glioma is commonly associated with neurofibromatosis type 1 (28), while hemangioblastoma is commonly associated with the VHL disease. The patient had the VHL disease with a germline mutation of the *VHL* gene. This fact strongly suggested that this optic nerve tumor is a hemangioblastoma.

Until now, 20 cases of optic nerve hemangioblastoma including this present case have been reported in the English literature (29–40). Fourteen of the 20 cases (70%) were associated with VHL. The ages of the 20 patients ranged from 15 to 64 years, with a mean being 34.8 years. There have been 13 females and 7 males diagnosed with this lesion. Nine of the patients had intraorbital masses, 5 patients had intracranial tumors, and 6 patients had a combination of intracranial and intraorbital masses. Meyerle et al. reported 9 cases reviewing the clinical course of retrobulbar hemangioblastomas including 4 optic nerve hemangioblastomas (35). In their series, 4 of 300 VHL patients developed optic nerve hemangioblastomas. From these data, the incidence of optic nerve hemangioblastomas is estimated as 1.3% in a population with VHL disease.

As complete surgical resection is curative, surgical resection is recommended as the first-line treatment for CNS hemangioblastomas. As an alternative or second-line treatment, stereotactic radiosurgery for hemangioblastomas has been indicated, especially for incompletely resected, residual, multiple, recurrent, and surgically inaccessible tumors that have anatomy unfavorable for surgical resection. Stereotactic radiosurgery is also indicated for patients with medical morbidities that make invasive procedures too risky and for VHL patients who have had multiple surgical resections. Stereotactic radiosurgery is able to target a small hemangioblastoma and offers the advantage of having a lower morbidity compared with surgical resection (41). In the past 20 years, stereotactic radiosurgery has been used as a minimally invasive adjuvant or salvage option for patients with hemangioblastomas. Before the era of stereotactic radiosurgery, management of residual or surgically inaccessible hemangioblastomas required the use of conventionally fractionated radiotherapy. Conventional radiotherapy has the disadvantage of exposing the surrounding normal tissue to irradiation and has the advantage of less adverse injury to neural tissues than non-fractionated stereotactic radiosurgery (42, 43). This patient was treated with 13 fractionated stereotactic radiosurgeries. However, despite a reduction in tumor volume, the patient lost her vision after the radiosurgery.

## Conclusion

Differential diagnosis for a solitary optic nerve tumor usually includes an optic nerve sheath meningioma, an optic schwannoma, and an optic glioma. However, in patients with VHL disease, hemangioblastoma should remain in the differential diagnosis of optic nerve tumors. The current report describes the first known case of an optic nerve hemangioblastoma treated with stereotactic radiosurgery. Optic nerve hemangioblastomas are extremely rare, and fractionated stereotactic radiosurgery can be occasionally indicated for treatment, when it is difficult to preserve vision with surgical resection. Although the approach resulted in tumor shrinkage, the patient lost eye sight. It is not clear whether the vision loss followed a natural course, as the patient presented with a visual acuity of 0.1, or radiosurgery somehow accelerated the process.

## Conflicts of interest

The authors declare no potential conflicts of interest with respect to this report, authorship and/or publication of this article.
